# Enrichment is not necessarily recruitment in root microbiome assembly

**DOI:** 10.1093/ismejo/wrag192

**Published:** 2026-07-21

**Authors:** Jingyin Xie, Tengxiang Lian

**Affiliations:** Guangdong Basic Research Center of Excellence for Precise Breeding of Future Crops, Guangdong Laboratory for Lingnan Modern Agriculture, Guangdong Provincial Key Laboratory for the Development Biology and Environmental Adaptation of Agricultural Organisms, South China Institute for Soybean Innovation Research, College of Agriculture, South China Agricultural University, No. 483 Wushan Road, Tianhe District, Guangzhou, Guangdong Province 510642, China; Guangdong Basic Research Center of Excellence for Precise Breeding of Future Crops, Guangdong Laboratory for Lingnan Modern Agriculture, Guangdong Provincial Key Laboratory for the Development Biology and Environmental Adaptation of Agricultural Organisms, South China Institute for Soybean Innovation Research, College of Agriculture, South China Agricultural University, No. 483 Wushan Road, Tianhe District, Guangzhou, Guangdong Province 510642, China

**Keywords:** root microbiome, rhizosphere enrichment, microbial recruitment, host-mediated selection, plant–microbe interactions

## Abstract

Rhizosphere enrichment is often interpreted as evidence that plants recruit beneficial microorganisms from soil, but enrichment and recruitment describe different levels of inference. Enrichment is a compositional pattern, whereas root association is a broader outcome produced by distinct processes through which microorganisms reach, attach to, colonize, and persist on roots. Within this outcome, recruitment in a narrower, directional sense denotes cases in which host-derived cues promote microbial attraction to, and/or specific attachment on, roots. Recruitment in this sense is independent of benefit because recruited microbes may be beneficial, neutral, or harmful. This distinction is often blurred when differential abundance alone is used to infer host-driven recruitment. Recruitment claims should instead be evaluated through microbial traits and host physiological, immune, and metabolic states that jointly determine root association. Motile bacteria with flagella, chemotaxis systems, and extracellular polysaccharides can respond directionally to root-derived cues and stabilize attachment on root surfaces. Many root-associated fungi lack comparable active motility, and their enrichment more often reflects contact opportunity, surface compatibility, and host immune permissiveness. Recent work further shows that enrichment can be uncoupled from plant benefit, because host physiological states may create permissive niches, whereas strain-level competition determines functional outcomes. We therefore propose a framework that separates microbe-driven attraction and attachment from host-mediated accommodation and persistence. Rhizosphere enrichment is thus an observable outcome, not a default signature of recruitment or benefit. Precise recruitment language requires trait-based, spatial, temporal, and functional evidence linking microbial presence to the mechanisms that generate and maintain root association.

## Recruitment has become a default interpretation of enrichment

The idea that plants recruit root-associated microbes has become an organizing concept in rhizosphere biology. Influential reviews and recent studies have portrayed the root as an active organizer that draws beneficial partners from surrounding soil [1–3]. The concept is now deeply embedded in the field, yet rhizosphere enrichment alone does not establish plant-mediated recruitment or benefit, because it may reflect abiotic filtering, niche competition, stochastic colonization, or altered host immunity rather than targeted host selection [4–6].

Yet recruitment is rarely defined in terms of the specific assembly process being inferred. In some studies, it refers to the chemotactic attraction of motile bacteria toward root-derived compounds, a process for which substantial molecular evidence exists [7, 8]. In other studies, recruitment denotes selective enrichment of taxa in amplicon sequencing datasets, or more broadly, functions as a rhetorical shorthand for any host-associated shift in community composition, even when there is no evidence that the enriched organisms were actively attracted [9–11]. These usages describe fundamentally different biological processes, but are often collapsed into a single term.

A similar contrast appears in the evidentiary basis of recent studies. Recent plant–microbiome studies often infer host-mediated selection or recruitment from amplicon-based compositional patterns, such as differentially enriched bacterial 16S rRNA gene amplicon sequence variants (ASVs) and fungal internal transcribed spacer ASVs across bulk soil and rhizosphere compartments or among host genotypes [9, 12]. By contrast, only a smaller group of studies provides direct mechanistic evidence, such as chemotaxis assays, structural mutant experiments, or live imaging of microbial movement toward roots [7, 13]. Taken together, this pattern suggests that recruitment is often used as a default interpretation of enrichment rather than as a conclusion grounded in process-level evidence.

The distinction of enrichment from recruitment matters because terminology shapes what counts as adequate evidence. Enrichment may arise through multiple non-exclusive processes. A microbe may be chemotactically attracted to the root, selectively tolerated by the host immune system [14, 15], competitively favored under root-modified conditions [16, 17], enriched because the host suppresses competing taxa [18], or driven by competition and metabolic cross-feeding among microbes that requires no host signal [19, 20]. These alternatives differ profoundly in biological meaning, yet they can generate similar compositional signatures. Among them, only chemotactic attraction represents a directional, host-cued route consistent with the sense of recruitment used here, although specific attachment can also support a recruitment claim when experimentally demonstrated. Selective tolerance and suppression of competing taxa are forms of host-mediated selection, competitive advantage under root-modified conditions reflects niche filtering, and microbial cross-feeding is a microbe–microbe effect. These remaining processes can generate enrichment without requiring the host to direct the microbe to the root. The plant immune system acts as a gatekeeper of community composition, but immune-mediated selection is mechanistically distinct from directional attraction [21, 22].

Root enrichment, in practice, does not always signal directional recruitment, nor does it guarantee benefit to the plant. For example, *Streptomyces* were repeatedly enriched in *Arabidopsis roots*, a pattern that could be read as stress-induced recruitment of beneficial microbes. However, mechanistic analyses linked this enrichment to drought-associated suppression of host iron uptake and salicylic-acid-related immunity, whereas plant benefit was uncoupled from enrichment [4]. The same uncoupling appears in the wheat rhizosphere, where seed-borne bacteria assemble the root community through microbe–microbe niche partitioning and facilitation rather than host-directed recruitment [23], and a fungal endophyte promotes root colonization by regulating host cell death, indicating that colonization can be enhanced through host-state manipulation after contact rather than requiring evidence of directional attraction [24]. Together, these examples show that enrichment can reflect host-created permissive niches or microbial competition, rather than directional host invitation or guaranteed plant benefit.

Recruitment has a broader and well-established meaning in ecology. In plant population and community ecology, for example, recruitment commonly refers to the addition or establishment of new individuals, such as seedlings, into a population or community [25, 26]. We do not seek to replace these broader ecological usages, nor do we argue that recruitment must always imply attraction, attachment, accommodation, persistence, and benefit. Our concern is narrower: in the root microbiome literature, recruitment is often inferred from abundance-based community profiles, where enrichment near roots is interpreted as active host-mediated acquisition from the surrounding soil. This inference requires clearer evidence about which assembly process is being invoked. In this Review, we ask whether bacterial-fungal structural differences help determine when recruitment is mechanistically justified. We compare the two kingdoms across three structural dimensions, use recent drought-associated *Streptomyces* work as an illustrative case, develop a framework that distinguishes microbe-driven from host-driven assembly processes, and outline predictions that may help shift the field from post hoc interpretation to hypothesis-driven investigation.

## Microbial traits constrain how organisms associate with roots

**Figure 1:**
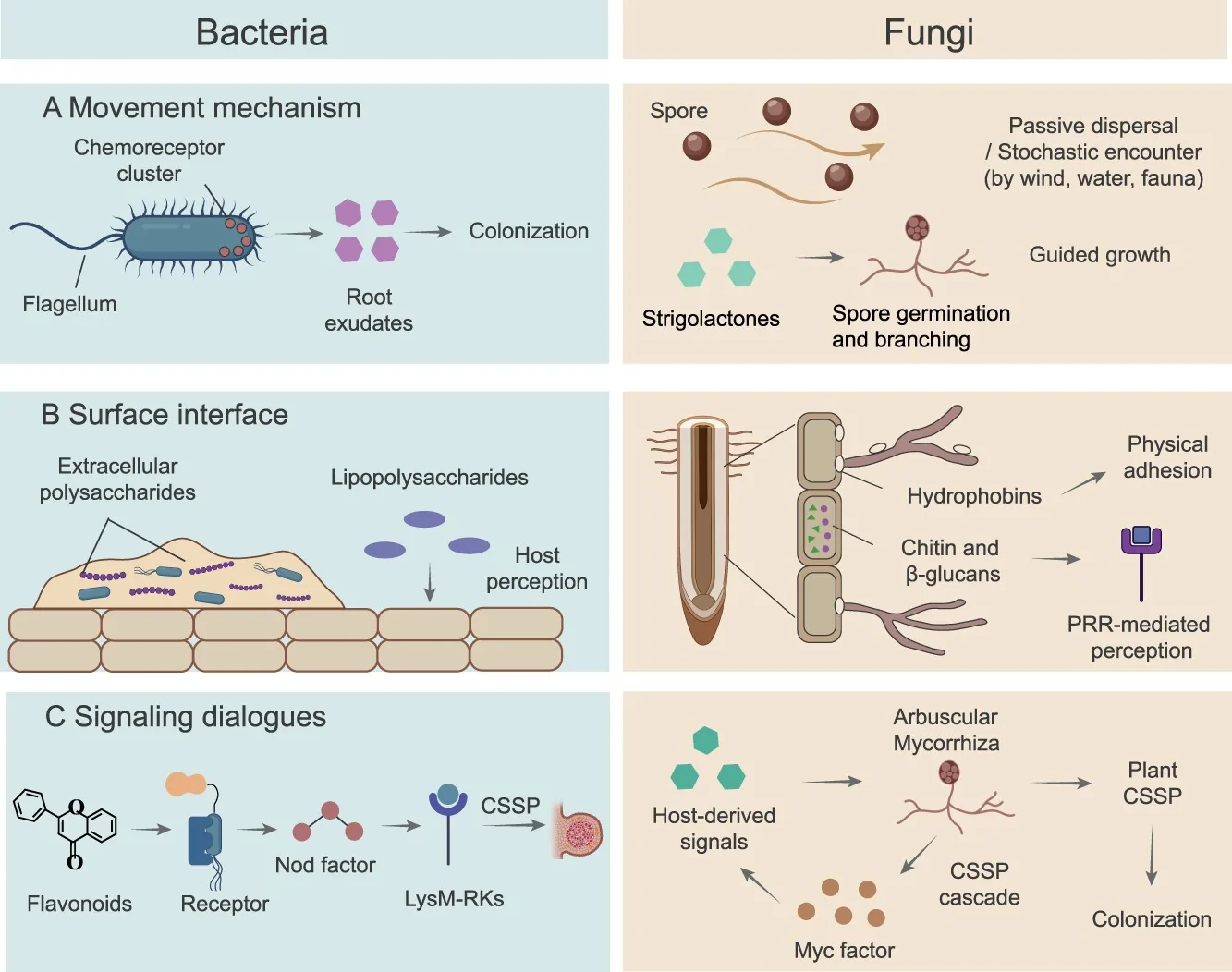
Structural and mechanistic divergence in bacterial and fungal root association. (a) Movement mechanism. Bacterial approach is often an active, microbe-driven process mediated by flagella and chemoreceptor clusters that perceive root-derived chemical gradients and guide directional movement. In arbuscular mycorrhizal fungi, strigolactones can stimulate spore germination, hyphal branching, and oriented hyphal growth, but this process represents guided growth rather than reversible motility-based chemotaxis. (b) Surface interface. At the root surface, bacteria commonly rely on EPSs to support attachment and biofilm formation, whereas lipopolysaccharides and other surface molecules influence host perception. In fungi, hydrophobins contribute to physical adhesion, whereas successful establishment depends more strongly on host gatekeeping. Fungal cell-wall components, such as chitin and β-glucans, are recognized through pattern-recognition receptor-mediated (PRR-mediated) perception, thereby influencing whether colonization is tolerated or restricted. (c) Signaling dialogues. In a limited number of co-evolved symbioses, reciprocal molecular signaling promotes colonization. In rhizobium–legume interactions, host flavonoids induce bacterial Nod factor production, and these signals are perceived by plant LysM-RKs, which activate the downstream CSSP. In arbuscular mycorrhiza, fungal Myc factors activate the plant common symbiosis signaling pathway, and host-derived strigolactones promote early fungal development. These signaling exchanges represent specialized forms of host accommodation rather than a general mechanism of recruitment. Some graphical elements were created with BioRender.com and BioGDP resources. BioRender citation: Created in BioRender. Jingyin, X. (2026) https://BioRender.com/1x3z04a. BioGDP attribution: Created with BioGDP.com.

Structural differences between bacteria and fungi shape not only how these organisms interact with plant roots, but also whether their enrichment can reasonably be interpreted as recruitment. Rather than cataloging these traits exhaustively, we focus here on three dimensions most relevant to that question. The first concerns how microbes reach the root. The second concerns what happens when they encounter the root surface. The third asks how far the interaction extends into reciprocal molecular dialogue. Considered together, these dimensions help clarify why similar compositional outcomes may arise from very different assembly processes (Fig. 1). We focus on bacteria and fungi because the structural contrast that bears on recruitment is best characterized in these two groups, namely active motility and chemotaxis in bacteria versus guided growth and surface compatibility in fungi. Archaea are also members of the root microbiome, and archaeal abundances can shift with root compartment and plant development [27]. However, the cellular basis of archaeal root colonization remains less resolved than for bacteria or fungi; where relevant approach or attachment mechanisms are defined, the same evidentiary logic developed here should apply.

### Directional approach depends on motility and growth mode

The most consequential structural difference between bacteria and fungi, in the context of recruitment, lies in how they approach the root. Many rhizosphere bacteria possess flagella and chemotaxis systems that enable directional movement along root exudate gradients on timescales of seconds to minutes [7, 8]. In these organisms, flagella provide motility, whereas chemosensory pathways convert exudate gradients into biased movement toward the root surface [28]. Disrupting flagellar or chemotaxis genes reduces root colonization in several well-studied systems, including *Pseudomonas fluorescens* and *Bacillus subtilis* [29, 30], indicating that directional movement is not merely correlated with root association but contributes directly to it. Likewise, chemotaxis receptor mutants of *Azospirillum brasilense* fail to preferentially colonize the root maturation and elongation zones that produce organic acid attractants, further showing that bacterial spatial positioning on roots is shaped by perception of host-derived signals [31]. More recent work in microbial chemotaxis and rhizosphere biology suggests that bacterial approach can involve sustained movement and collective gradient formation rather than a single encounter event [32–34]. Cells that have already reached the root surface can amplify directional migration by releasing secondary attractants, and bacteria may continue to reposition as roots grow and immunologically active zones shift over time [8, 35, 36]. In at least some bacterial systems, then, root association is mechanistically compatible with the language of recruitment because the microbe possesses a structural means of directed approach.

Directional attraction, however, does not explain enrichment uniformly even within bacteria. Some Actinobacteria are repeatedly enriched in root-associated communities even though flagellar motility is absent or poorly characterized in many lineages [37, 38]. Their recurrent enrichment in the rhizosphere cannot be explained by directional attraction alone. Even within bacteria, therefore, recruitment is not a universally valid interpretation, and the mechanism underlying enrichment must be evaluated case by case.

For most root-associated fungi, the situation is fundamentally different. These organisms generally lack flagella and do not perform chemotaxis, although two important boundary cases complicate this contrast: arbuscular mycorrhizal fungi (AMF) and zoosporic oomycetes [39, 40]. In the dominant fungal groups associated with roots, propagules are dispersed by wind, water, or soil fauna, so the initial encounter between a fungal propagule and a root is often stochastic from the host’s perspective [41]. The best-known exception is the response of AMF to host-derived strigolactones. In this case, strigolactones stimulate spore germination, hyphal branching, and pre-symbiotic hyphal development near the root [42, 43]. Yet even here, guided hyphal growth differs from reversible bacterial swimming. We therefore suggest that this process is better described as guided growth than as motility-based attraction. This distinction marks a real difference in the physical and energetic basis of host approach, and therefore in how root enrichment should be interpreted. Zoosporic oomycetes such as *Phytophthora* spp. provide a second boundary case because they produce flagellated zoospores capable of chemotactic movement toward root exudates [40]. Although oomycetes are phylogenetically distinct from true fungi, they are often grouped with them in community studies. Their existence reinforces an important point. The key distinction for recruitment is not taxonomic identity, but structural capacity. What matters is whether an organism can approach the root directionally, not simply which kingdom it belongs to.

### Root surface colonization depends on attachment and host permissiveness

Reaching the vicinity of the root is only the beginning. Whether colonization proceeds depends on what happens at the interface between microbial surface and host tissue. In bacteria, extracellular polysaccharides (EPSs) promote adhesion to root surfaces and support biofilm formation [7], whereas lipopolysaccharides influence how the host perceives the microbe [44]. Some root-derived compounds coordinate multiple stages of colonization at once. In the cucumber–*Bacillus velezensis* system, for example, D-galactose stimulates both chemotaxis and biofilm formation, indicating that the same host cue can guide bacterial movement at a distance and stabilize attachment at close range [45]. Consistent with this, genetic studies in several bacterial systems link extracellular polymer production, surface structures and biofilm traits to root colonization, indicating that attachment can be experimentally separated from initial approach [46].

In fungi, the decisive issue at the root surface is less often whether the organism can attach than whether the host permits it to remain. Hydrophobins on the hyphal surface contribute to physical adhesion to plant tissues, but they do not direct growth toward the root [47]. Once contact is established, colonization depends heavily on how the host interprets fungal cell-wall components such as chitin and β-glucans [48, 49]. In mycorrhizal systems, the common symbiosis signaling pathway (CSSP) enables host accommodation of intracellular colonization [50], and AMF can further stabilize this interaction by deploying effector proteins such as Nuclear Localized Effector1, a *Rhizophagus irregularis* nuclear-localized effector that interferes with histone H2B mono-ubiquitination and helps promote mycorrhizal colonization [51]. In many non-mycorrhizal cases, however, the same interface instead determines whether the fungus is tolerated or excluded [19]. This balance is dynamic rather than fixed. Host immune output shifts with colonization density and with the broader microbial context [14, 52]. For fungi, then, the critical question is often not whether the organism can locate the root, but whether the host environment allows it to stay. These examples support the interpretation that fungal enrichment can reflect accommodation and filtering rather than recruitment in the strict directional sense [24, 49, 50].

### Reciprocal signaling is limited to specialized interactions

In a limited number of well-characterized symbioses, plant–microbe interactions extend beyond physical approach and surface contact to include reciprocal molecular signaling. The rhizobium–legume association provides the clearest example of this deeper level of dialogue. In this system, host-derived flavonoids stimulate bacterial Nod factor production, and these Nod factors are then perceived through lysin motif (LysM) receptor kinases (RKs), initiating the signaling cascade that culminates in nodule organogenesis [53]. A related logic operates in arbuscular mycorrhiza. There, fungal Myc factors, which are lipo-chitooligosaccharides, activate the plant CSSP, and host-derived strigolactones reinforce the interaction by promoting the earliest stages of fungal development and colonization. Together, strigolactones and Myc factors form a positive feedback loop that stabilizes the establishment of symbiosis [50, 54, 55]. These systems show that genuine molecular dialogue between host and microbe does occur and, in a restricted set of co-evolved interactions, can actively facilitate colonization.

Reciprocal signaling of this kind has not, however, been demonstrated for most plant–bacterium or plant–fungus associations. In many bacterial cases, the available evidence points instead to a broader strategy of immune modulation after contact has already been made. Recent work shows that a substantial fraction of root-associated bacterial commensals can suppress microbe-associated molecular pattern-triggered immunity in *Arabidopsis* [14, 56]. Beneficial bacteria can achieve this through several mechanisms, including acidification of the surrounding environment [57] and modulation of host immune outputs by commensal bacterial communities [56]. These activities clearly influence whether colonization succeeds, but they do not by themselves demonstrate directional recruitment. Rather, they are better understood as strategies of accommodation or immune evasion that operate after the bacterium has already reached the root surface.

A similar distinction applies in fungi. AMF can deploy effector proteins that suppress host defense responses and stabilize colonization after contact has been established [51]. In other words, they shape the outcome of colonization rather than the initial process by which the fungus arrives at the root. More broadly, reciprocal molecular signaling appears to be a defining feature of a limited set of co-evolved symbioses rather than a general property of rhizosphere assembly. For most plant-associated microbes, the existence of signaling after contact should therefore not be taken as evidence that recruitment, in the directional sense, has already occurred.

## Enrichment does not identify the process that produced it

Bacteria and fungi associate with roots through different causal routes. This divergence extends from modes of approach to surface polymers and signal-transduction architectures. In bacteria, root association may begin with directional movement toward exudate gradients and proceed through attachment to the root surface [29, 58, 59]. In fungi, colonization more often depends on contact opportunity, surface compatibility, and host immune acceptance [24, 51, 60, 61]. Treating both pathways as recruitment therefore compresses distinct causal processes into a single word. Members of both bacterial and fungal communities contribute to plant health, disease suppression, and responses to developmental shifts in root exudation [59, 62, 63]. The problem is not that one kingdom matters more than the other, but that the mechanisms underlying enrichment are not the same. Using one term for both can obscure where causal agency resides and prevent the field from asking the right mechanistic questions in each system.

### Assembly mechanisms differ in where causal agency resides

**Figure 2:**
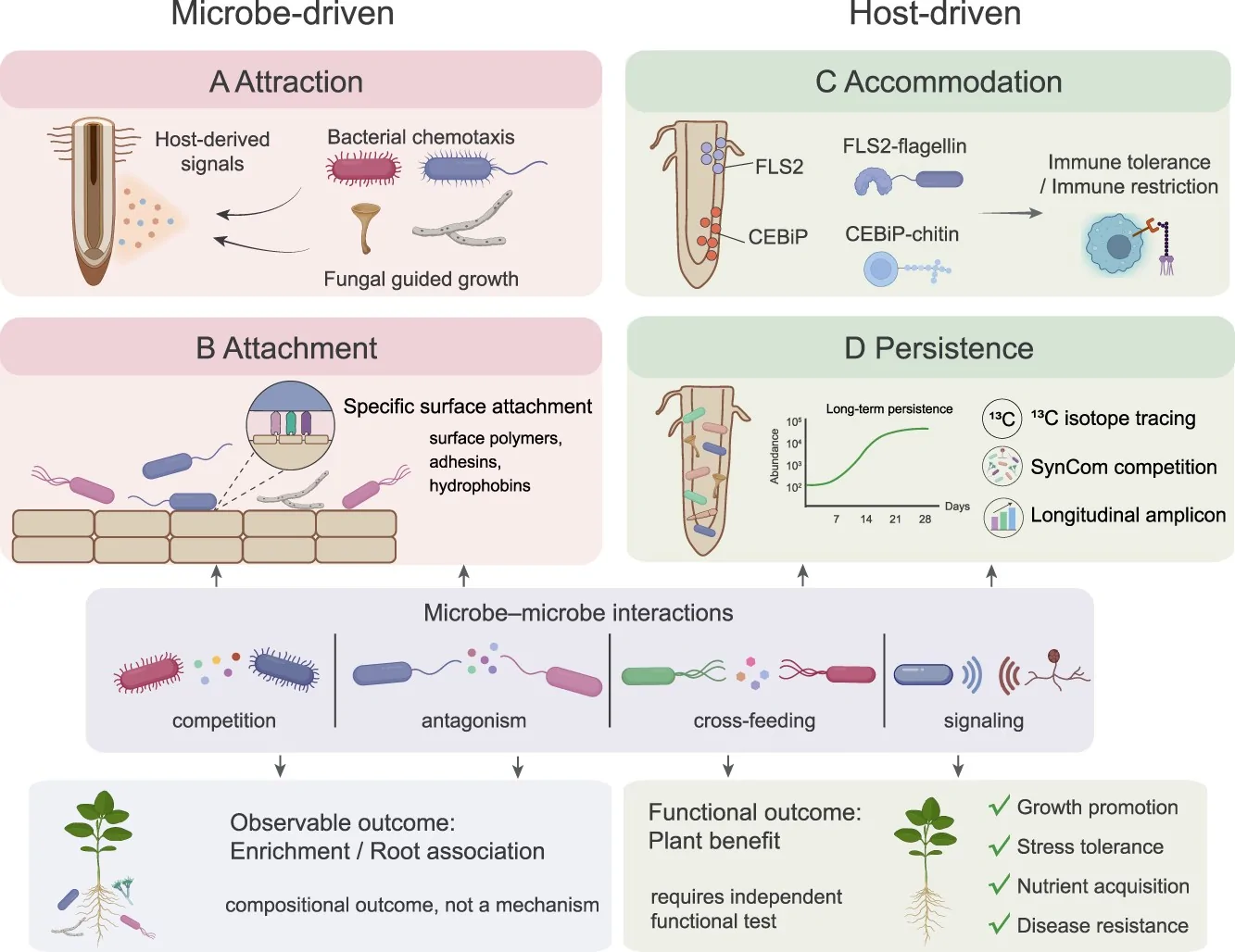
Four-process framework distinguishing directional recruitment, host-mediated selection, microbe–microbe interactions, enrichment, and plant benefit. Root association can arise through four alternative and potentially overlapping processes. **Attraction** and **attachment** are mainly microbe-driven, referring respectively to directional approach toward host-derived cues and specific surface association with the root. **Accommodation** and **persistence** are mainly host-driven, referring to host permissiveness for colonization and sustained microbial presence over time. Accommodation may involve PRRs, including flagellin-sensitive 2 for flagellin perception and chitin elicitor-binding protein for chitin perception. Persistence can be assessed using carbon-13 isotope tracing, SynCom competition, and longitudinal amplicon profiling. Microbe–microbe interactions, including competition, antagonism, cross-feeding, and signaling, can influence all four processes. Enrichment/root association is an observable compositional outcome, whereas plant benefit is a separate functional outcome requiring independent testing. Some graphical elements were created with BioRender.com and BioGDP resources. BioRender citation: Created in BioRender. Jingyin, X. (2026) https://BioRender.com/1x3z04a. BioGDP attribution: Created with BioGDP.com.

To address this problem, we propose that rhizosphere enrichment and root association can be understood through four processes, organized by where causal agency resides (Fig. 2). Two are primarily microbe-driven and two are primarily host-driven. We treat these as alternative and potentially overlapping routes to enrichment rather than as obligatory stages of recruitment; not every root association passes through all four. The first microbe-driven process is attraction, which we define as directional movement or growth toward host-derived signals. Evidence for attraction must demonstrate directionality, because enhanced growth in the presence of exudates does not by itself qualify as attraction unless that growth is oriented toward the signal source. In bacteria, attraction is mediated by flagellar motility and chemotaxis signaling pathways and can be tested with microfluidic chemotaxis assays [28, 31, 64]. In fungi, the corresponding question is whether hyphal growth is oriented toward host cues. This can be examined using split-root systems, directional cue application or chemotropism assays, although the slower timescale and limited reversibility of hyphal growth make interpretation more complex [54, 65, 66]. The second microbe-driven process is attachment, which we define as physical association with the root surface through specific molecular interactions rather than passive deposition. This process can be tested by comparing wild-type organisms with structural mutants lacking EPSs, flagella, or hydrophobins, thereby distinguishing specific binding from nonspecific contact [46, 67–69].

On the host side, we distinguish two additional processes. Accommodation denotes host-mediated permissiveness, including immune tolerance, nutrient provisioning, or tissue modification that allows colonization to proceed. It is best tested by manipulating the host rather than the microbe. For example, immune mutants with altered perception of microbial cell-wall components, including chitin perception mutants, can reveal whether host tolerance is necessary for colonization [61, 70]. Evidence that root-associated commensals actively suppress pattern-triggered immunity further indicates that accommodation is a widespread biological process rather than a passive default state [14, 56]. These experiments depend on gnotobiotic systems with defined microbial inputs, ranging from single-strain inoculation to reduced synthetic communities, because only such systems can cleanly connect specific host genotypes to specific colonization outcomes [17, 71–73]. The second host-driven process is persistence, defined as sustained presence and proliferation over time rather than transient colonization. Persistence cannot be inferred from a single time point. It requires time-resolved evidence, such as ^13^C isotope tracing or stable-isotope-informed approaches that distinguish metabolically active colonizers from dormant cells [74, 75], synthetic community (SynCom) competition experiments that reveal whether structural traits affect long-term competitive outcomes [17, 20], or longitudinal amplicon profiling that tracks assembly across development [27, 76, 77].

Microbe–microbe interactions cut across all four processes (Fig. 2). Competition, antagonism, metabolic exchange, and intermicrobial signaling can influence attraction through signal modification or quenching; attachment through coaggregation or exclusion from biofilms; accommodation by altering the immune context experienced by neighboring taxa; and persistence through longer-term competitive outcomes [17, 19, 20]. These interactions therefore represent an alternative route by which enrichment can arise without directional host recruitment. They should be considered, and where possible experimentally tested or controlled for, before enrichment is attributed to host-driven recruitment.

Within this framework, enrichment is the compositional pattern captured by sequencing, namely a change in the relative or absolute abundance of a taxon near the root compared with bulk soil [59, 78]. It is an outcome rather than a mechanism. The broader outcome in which microorganisms reach, attach to, colonize, and persist on roots is what we term root association. Directional recruitment, in the sense used here, is the narrower claim that host-derived cues promote attraction and/or specific attachment, and requires evidence beyond compositional change; accommodation and persistence can also produce enrichment and stable root association, but on their own are better described as host-mediated selection, permissiveness, or filtering than as recruitment.

Plant benefit is a further functional claim: it requires evidence that the enriched organism improves host performance, not merely that it becomes more abundant. A given taxon may be beneficial, neutral, or detrimental depending on host genotype, environmental conditions, microbial dose, and community context, and its net effect may reverse across these variables [52, 59, 73, 79]. Because attraction, attachment, accommodation, and persistence can occur without conferring benefit, and benefit can arise without directional recruitment, benefit constitutes an independent axis of evidence. It should be tested under defined conditions using functional or phenotypic assays, such as gnotobiotic or synthetic-community experiments comparing host performance with and without the focal microbe.

### Canonical systems reveal different routes to enrichment

Several systems show that enrichment can be uncoupled from directional recruitment, plant benefit, or both (Table 1). In reconstituted *Arabidopsis* root communities, an enriched bacterial consortium suppressed root growth until a *Variovorax* lineage relieved the inhibition [71]. Rhizosphere metabolite dynamics can also favor microbes with matching substrate preferences [63]. Similarly, disease-suppressive microbiomes can emerge through community-level restructuring rather than attraction of a single beneficial taxon [62]. These examples separate what produces enrichment, whether recruitment occurred, and whether the outcome benefits the host.

**Table 1:** Examples illustrating that enrichment, recruitment, and benefit require different evidence.

**Category**	**System or example**	**Observed pattern**	**Inference requiring caution**	**Alternative process highlighted**	**Reference**
Stress-associated bacterial enrichment	Drought-induced *Streptomyces* in Arabidopsis roots	*Streptomyces* lineages are enriched under drought.	Enrichment under drought does not by itself indicate host recruitment of beneficial Streptomyces.	Host iron and salicylic-acid-related immune state, together with strain-level competition.	[4]
Stress-associated bacterial or fungal shifts	Drought-associated microbial shifts in grass and sorghum roots	Drought alters root-associated microbial community composition.	Compositional change under drought does not by itself demonstrate directional recruitment.	Host physiological filtering, drought-created niches, and selective or stochastic assembly.	[6, 37]
Exudate-mediated filtering	Rhizosphere metabolite preferences	Root-associated taxa are enriched according to substrate use.	Substrate-based enrichment does not by itself indicate host-directed recruitment.	Exudate-driven substrate filtering and growth of microbes already able to use available metabolites.	[63]
Host chemical filtering	Coumarin-mediated reshaping of the Arabidopsis root microbiota	Taxa are enriched or depleted under coumarin-related conditions.	Coumarin-associated shifts do not by themselves indicate active recruitment of specific taxa.	Selective inhibition or facilitation under iron-related chemical conditions.	[18]
Host immune and nutrient filtering	Salicylic-acid and phosphate-linked root microbiota assembly	Immune or nutrient-signaling mutants show altered root microbiota composition.	Altered composition in immune or nutrient mutants does not by itself establish directional recruitment.	Host immune and nutrient filtering after microbial encounter.	[21, 72]
Intermicrobial metabolism	Bacterial exometabolism cofunctioning in root microbiota establishment	Commensal establishment depends on community context.	Community-dependent establishment does not by itself indicate host recruitment.	Bacterial cross-feeding and cofunctioning among community members.	[20]
Intermicrobial assembly and seed origin	Seed-borne bacteria in the wheat rhizosphere	Seed-derived taxa contribute to rhizosphere assembly.	Rhizosphere enrichment does not by itself indicate recruitment from the surrounding soil.	Seed origin and microbe–microbe niche partitioning and facilitation.	[23]
Benefit uncoupling	Reconstituted Arabidopsis synthetic communities with Variovorax	Plant growth outcome depends on community composition and lineage identity.	Enrichment or abundance alone does not establish plant benefit.	Lineage-specific function and community context.	[71]
Fungal host accommodation	Root-colonizing fungal endophyte manipulating host cell death	Fungal colonization increases under altered host cell-death state.	Colonization does not by itself establish directional recruitment.	Host accommodation or host-state manipulation after contact.	[24]
Archaeal and underexplored groups	Archaeal shifts in rice root-associated microbiota	Archaeal taxa vary by root compartment and plant development.	Compartment- and stage-related variation does not by itself indicate directional archaeal recruitment.	Detectable root association with unresolved approach, attachment, and filtering mechanisms.	[27]

Stress-associated enrichment is frequently interpreted through the cry-for-help hypothesis, in which stressed plants reshape their microbiome to increase protective microbes. Our argument does not reject this framework, but separates its evidentiary claims: stress-induced compositional change is enrichment; active host direction is directional recruitment; and improved host performance is benefit. These propositions may coincide, but they need not. Drought-induced *Streptomyces* enrichment illustrates this distinction: repeated enrichment under drought could suggest recruitment of beneficial helpers, yet recent work linked enrichment to host iron and immune status, whereas plant benefit remained uncoupled from enrichment and depended on strain-level interactions [4]. Thus, the framework proposed here asks which component has been demonstrated in a given system: stress-induced enrichment, host-directed recruitment, microbial benefit, or a combination of all three.

The value of this framework becomes clearer when applied to well-studied systems. In the *Pseudomonas* root association, experimental evidence supports chemotaxis toward root exudates, a role for flagella in root colonization, and EPS-mediated biofilm formation on root surfaces [45, 46, 80]. In arbuscular mycorrhizal symbiosis, strigolactones stimulate pre-symbiotic fungal development near roots, a fungal form of approach discussed above as guided growth rather than motility-based attraction [43, 81]. At the same time, the CSSP mediates host accommodation and permits intracellular fungal establishment [61, 70]. Recruitment is therefore only partly applicable in this system, a more precise description would be host-guided colonization accompanied by active accommodation. By contrast, amplicon-based fungal shifts of root-associated fungi can identify enrichment, but not resolve whether the underlying process is attraction, host filtering, priority effects, or persistence after contact [6, 59, 79]. Microbe–microbe competition or host filtering may equally account for such patterns [17, 19]. In such cases, these taxa are more appropriately described as enriched rather than recruited, and the mechanism underlying their enrichment should remain open to experimental testing.

### Testable predictions can separate attraction from accommodation

Predictions from this framework should begin with the step being tested. Attraction predicts directional approach before stable colonization. In motile bacteria, this can be examined by comparing wild-type strains with chemotaxis or motility mutants under matched inoculum density, then measuring movement toward root exudates, early root arrival, and later enrichment. Loss of early approach would support attraction as the mechanism, whereas unchanged arrival followed by altered abundance would point to attachment, accommodation, or persistence [7, 8, 30]. This design keeps differential abundance from carrying more mechanistic meaning than it can support.

Because fungal approach proceeds by guided growth rather than chemotaxis, this test must be adjusted for fungi. Hyphal responses to strigolactones or root exudate fractions support attraction only when growth is oriented toward a signal source and precedes host entry. Hydrophobins or other surface traits are expected to change contact stability, with weaker predictions for long-distance approach [67]. If fungal abundance changes mainly with host chitin perception, symbiosis signaling, or immune tone, the result is more consistent with accommodation than attraction [61, 70]. This distinction prevents fungal enrichment from being interpreted through a bacterial model of movement.

Accommodation predicts that host immune or nutrient perturbations alter abundance even when microbial approach traits remain intact. Drought-induced *Streptomyces* enrichment fits this logic because manipulation of salicylic-acid-related immunity and iron availability changed *Streptomyces* abundance, whereas enrichment and plant benefit remained separable [4]. Tests with chitin receptors, the ENHANCED DISEASE SUSCEPTIBILITY 1–PHYTOALEXIN DEFICIENT 4 immune signaling node, or downstream pattern-triggered immunity can locate the host gate at ligand perception, immune state, or nutrient-linked niche conditions [14, 15, 61].

Root exudate mutants become most informative when paired with host-state manipulation. A bacterial shift after loss of an exudate class supports attraction only if the affected taxa also show directional responses to that compound. Without this link, the same community shift could reflect altered pH, iron mobilization, carbon availability, or immune status [18, 57, 82, 83]. For fungi, exudates may change the local niche without serving as directional cues. Factorial designs that vary exudate chemistry and immune or nutrient state independently are therefore needed to assign enrichment to attraction or accommodation.

Temporal and spatial measurements can connect these perturbations to process. Attraction should be visible early, before growth and competition reshape abundance, and should place responsive bacteria near exudate-release zones. Accommodation is expected to appear after contact, as successful lineages persist in regions where immune activity, nutrient availability, or surface chemistry permit colonization. Live imaging, rhizosphere metabolic imaging, spatially resolved host transcriptomics, and absolute abundance measurements can test these sequences in intact roots [64, 84, 85]. These approaches are most informative when paired with systems in which host-derived compounds, microbial sensing or motility traits, and spatial colonization outcomes have been experimentally linked [13, 29, 31, 45]. A prediction is informative when it separates timing, location, and abundance. A single endpoint composition profile cannot do that.

Together, these predictions distinguish mechanism. Loss of early root approach in chemotaxis mutants supports attraction, whereas altered colonization without impaired approach supports accommodation. Mixed outcomes identify where attraction, attachment, accommodation, and persistence overlap. Recruitment should be used only when experiments identify the process producing enrichment.

### Recruitment claims require evidence beyond differential abundance

A minimum evidentiary standard follows from this framework. Studies that claim recruitment should provide at least one independent line of evidence at the level of attraction or attachment, such as chemotaxis assays, colonization experiments with structural mutants, or live imaging of directional approach [8, 64, 68]. Correlations between host genotype and microbial composition are informative, but not sufficient on their own, because they cannot distinguish attraction from accommodation or from environmental filtering. Genome-wide association studies linking host loci to microbial community composition likewise provide valuable insight into host-mediated selection, yet they do not by themselves reveal whether host effects operate through exudate-mediated attraction, immune-mediated accommodation, or nutrient-mediated competitive advantage [9, 79].

Emerging tools may help disentangle these processes. Rhizosphere metabolic imaging can map root exudates and microbial metabolites directly at plant–microbe interfaces [86, 87], and genome-resolved metatranscriptomics can link microbial activity states with colonization determinants [88]. Trait databases for motility, chemotaxis, and guided growth could help prioritize enriched taxa for targeted assays. Constraint-based community metabolic models, adapted from human microbiome research, may further clarify how carbon partitioning and cross-feeding shape microbiome assembly and predict competitive persistence outcomes [89, 90]. More broadly, recruitment claims need standardized evidence criteria. By analogy with Koch’s postulates, which linked microbial presence to causal evidence, compositional enrichment alone is insufficient. Directional recruitment should require evidence of approach or specific attachment; host-directed recruitment should show that host genotype or physiology alters colonization with microbial approach traits are controlled; and beneficial recruitment should include an independent host performance test.

## Concluding remarks

The plant microbiome literature has widely adopted recruitment as a default explanation for rhizosphere enrichment, yet the same compositional outcome can arise through different causal pathways. Microbe-driven processes such as attraction and attachment are distinct from host-driven processes such as accommodation and persistence, and these should not be treated as interchangeable explanations. Enrichment is what amplicon sequencing captures. It should not be mistaken for the mechanism that produced it.

The implications extend well beyond terminology. If enrichment continues to be equated with recruitment, and recruitment with benefit, the field risks detecting patterns without resolving the processes or functional consequences that underlie them. A more precise vocabulary makes it possible to ask whether enrichment depends on microbial motility or host immune tolerance, on structural interactions at the root surface or on competitive persistence, and on attraction, release from inhibition, or accommodation as the dominant axis of host control. Cross-kingdom microbiome analyses may obscure important causal differences by forcing both groups into a unified assembly narrative. This distinction also matters for microbiome engineering, because enhancing bacterial colonization may require optimization of chemotactic signaling and surface attachment, whereas promoting fungal association may depend more on modulating host immune thresholds and permissive host states.

The goal of this review is to restrict the use of recruitment to cases in which mechanistic evidence supports it, rather than to discard it as a concept. Treating recruitment as a testable hypothesis is necessary if plant microbiome research is to move beyond describing who is present and begin explaining how organisms arrived, why they persisted, and whether their presence matters for the host.

## Data Availability

Data sharing is not applicable to this article as no datasets were generated or analyzed during the current study.
